# BECN1 promotes radiation-induced G2/M arrest through regulation CDK1 activity: a potential role for autophagy in G2/M checkpoint

**DOI:** 10.1038/s41420-020-00301-2

**Published:** 2020-08-05

**Authors:** Ruixue Huang, Shanshan Gao, Yanqin Han, Huacheng Ning, Yao Zhou, Hua Guan, Xiaodan Liu, Shuang Yan, Ping-Kun Zhou

**Affiliations:** 1 Department of Occupational and Environmental Health, Xiangya School of Public Health, Central South University, 410078 Changsha, Hunan Province China; 2 Department of Radiation Biology, Beijing Key Laboratory for Radiobiology, Beijing Institute of Radiation Medicine, AMMS, 100850 Beijing, China; 3 Institute for Chemical Carcinogenesis, State Key Laboratory of Respiratory, School of Public Health, Guangzhou Medical University, 511436 Guangzhou, P. R. China

**Keywords:** Macroautophagy, Cancer prevention

## Abstract

Authophagy and G2/M arrest are two important mechanistic responses of cells to ionizing radiation (IR), in particular the IR-induced fibrosis. However, what interplayer and how it links the autophagy and the G_2_/M arrest remains elusive. Here, we demonstrate that the autophagy-related protein BECN1 plays a critical role in ionizing radiation-induced G_2_/M arrest. The treatment of cells with autophagy inhibitor 3-methyladenine (3-MA) at 0–12 h but not 12 h postirradiation significantly sensitized them to IR, indicating a radio-protective role of autophagy in the early response of cells to radiation. 3-MA and BECN1 disruption inactivated the G_2_/M checkpoint following IR by abrogating the IR-induced phosphorylation of phosphatase CDC25C and its target CDK1, a key mediator of the G_2_/M transition in coordination with CCNB1. Irradiation increased the nuclear translocation of BECN1, and this process was inhibited by 3-MA. We confirmed that BECN1 interacts with CDC25C and CHK2, and which is mediated the amino acids 89–155 and 151–224 of BECN1, respectively. Importantly, BECN1 deficiency disrupted the interaction of CHK2 with CDC25C and the dissociation of CDC25C from CDK1 in response to irradiation, resulting in the dephosphorylation of CDK1 and overexpression of CDK1. In summary, IR induces the translocation of BECN1 to the nucleus, where it mediates the interaction between CDC25C and CHK2, resulting in the phosphorylation of CDC25C and its dissociation from CDK1. Consequently, the mitosis-promoting complex CDK1/CCNB1 is inactivated, resulting in the arrest of cells at the G_2_/M transition. Our findings demonstrated that BECN1 plays a role in promotion of radiation-induced G2/M arrest through regulation of CDK1 activity. Whether such functions of BECN1 in G2/M arrest is dependent or independent on its autophagy-related roles is necessary to further identify.

## Introduction

Radiotherapy is a widely used strategy for the treatment of cancer patients. However, despite major advances in radiotherapy, the radioresistance of tumors remains the leading obstacle to their clinical treatment because it results in radiotherapy failure or tumor recurrence^1^. Approximately 10–45% of cancers are resistant to radiation, which greatly influences the outcomes of radiotherapy.

Autophagy is a process of cellular self-degradation that plays a critical role in maintaining the balance between cell survival and cell death^2^. Recent studies have indicated that the two roles of autophagy in cancer cells are associated with the initiation of a cascade of signaling pathways involving multiple molecules and transcription factors^3,4^. The suppression of BECN1 phosphorylation reduces autophagy in prostate cancer cells and thereby overcomes the radioresistance of these cells^5^. Conversely, the autophagy-mediated degradation of p62 and the c-Jun-mediated expression of BECN1 increase the radioresistance of lung cancer cells^6^. We established BECN1-knockout human triple-negative breast cancer (TNBC) MDA-MB-231 cells using the CRISPR/Cas9 system, and functional analyses revealed that BECN1 deficiency suppressed MDA-MB-231 cell proliferation by inducing arrest of the cell cycle at the G_1_ phase in vitro^7^, which suggested that BECN1 regulates the cell cycle.

The activation of cell-cycle checkpoints is another fundamental process for controlling cellular homeostasis during exposure to radiation. During the cell cycle, various checkpoints play an important role in monitoring and regulating the progression as well as the genomic stability in response to DNA damage or other stresses. Li et al. reported that radiation induces G_2_/M phase arrest in primary human renal clear cell carcinoma cells, which might contribute to radioresistance^8^. Carruthers et al. found that the suppression of ATM kinase, an upstream kinase of the G_2_/M checkpoint signal pathway involving CHK2/CDC25C/CDK1, by the inhibitor KU-55933 inactivates the G_2_/M checkpoint and radiosensitizes glioblastoma stem-like cells^9^.

**Fig. 1:**
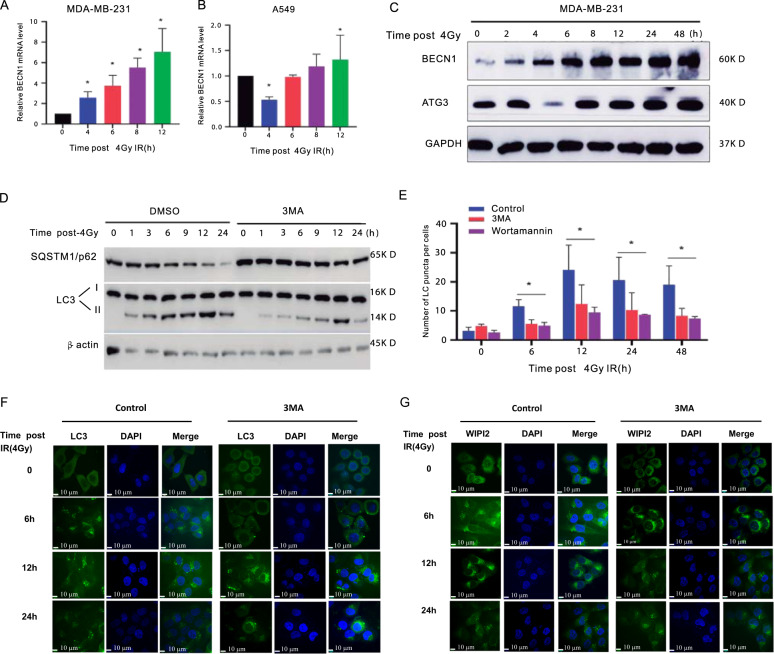
BECN1 was overexpressed in MDA-MB-231, A549, and mouse tissues post-exposure of IR. **a** qRT-PCR was performed to detect BECN1 mRNA expression and the asterisk (*) indicates a significant increase post 4 Gy IR exposure compared to the non-IR exposure in MDA-MB-231 cells. **b** qRT-PCR was performed to detect BECN1 mRNA expression and the asterisk (*) indicates a significant increase post 4 Gy IR exposure compared to the non-IR exposure in A549 cells. **c** Western blot assay was performed to detect theATG3 and BECN1 expression in MDA-MB-231 cells (*n* = 5) post 4 Gy IR exposure. **d** The protein levels of LC3 and SQSTM1/P62 in MDA-MB-231 cells were detected by Western blotting analysis. GAPDH was used as the loading control. MDA-MB-231 cells cotreated or not cotreated with 3-MA were harvested at the indicated timepoints after 4-Gy γ-ray-irradiation. **e** MDA-MB-231 cells cotreated or not cotreated with 3-MA or wortamannin were harvested at the indicated timepoints after 4-Gy γ-ray-irradiation. The number of LC3 puncta per cell. was analyzed and the asterisk (*) indicates a significant change compared with control group. **f** Autophagic GFP-LC3 puncta in 4-Gy γ-ray-irradiated MDA-MB-231 cells cotreated with or without 3-MA were detected by fluorescence confocal microscopy(bars: ×20 magnification). **g** Autophagic WIPI2in 4-Gy γ-ray-irradiated MDA-MB-231 cells cotreated with or without 3-MA was detected by fluorescence confocal microscopy(bars: ×20 magnification). The data are presented as the means ± SDs from three independent experiments *p < 0.01 compared with the control group.

In the present study, we focused on BECN1 based on the following considerations. BECN1, the mammalian homolog of yeast Atg6 and a highly conserved eukaryotic protein^10^, is a core autophagy-related protein that regulates vesicle nucleation during autophagosome formation. The blockage of autophagy using the autophagy inhibitor 3-methyladenine (3-MA) increases the number of cells in the G_2_/M state^11^. BECN1 also associates with class III phosphoinositide 3-kinase (PI3K) to promote the generation of phosphatidylinositol 3-phosphate^12^. BECN1 is also involved in the cellular response to radiation-induced damage or sensitivity^13^. Moreover, our previous study showed that the knockout of BECN1 suppresses TNBC cell proliferation and colony formation by inducing cell-cycle arrest at the G_1_ phase^7^. Our pilot study revealed that the inhibition of autophagy by 3-MA decreased the number of LC3 puncta and LC3B protein expression in γ-ray-irradiated cells (Fig. 1a). Thus, we hypothesized that autophagy-related BECN1 might play a markedly more important role in the activation of cell-cycle checkpoints in response to irradiation. Our findings demonstrated that BECN1 plays a role in promotion of radiation-induced G2/M arrest through regulation of CDK1 activity. Whether such functions of BECN1 in G2/M arrest is dependent or independent on its autophagy-related roles is necessary to further identify.

## Results

### BECN1 protein was remarkably upregulated in A549 cells, Hela cells, and mice lung fibrosis tissues

**Fig. 2:**
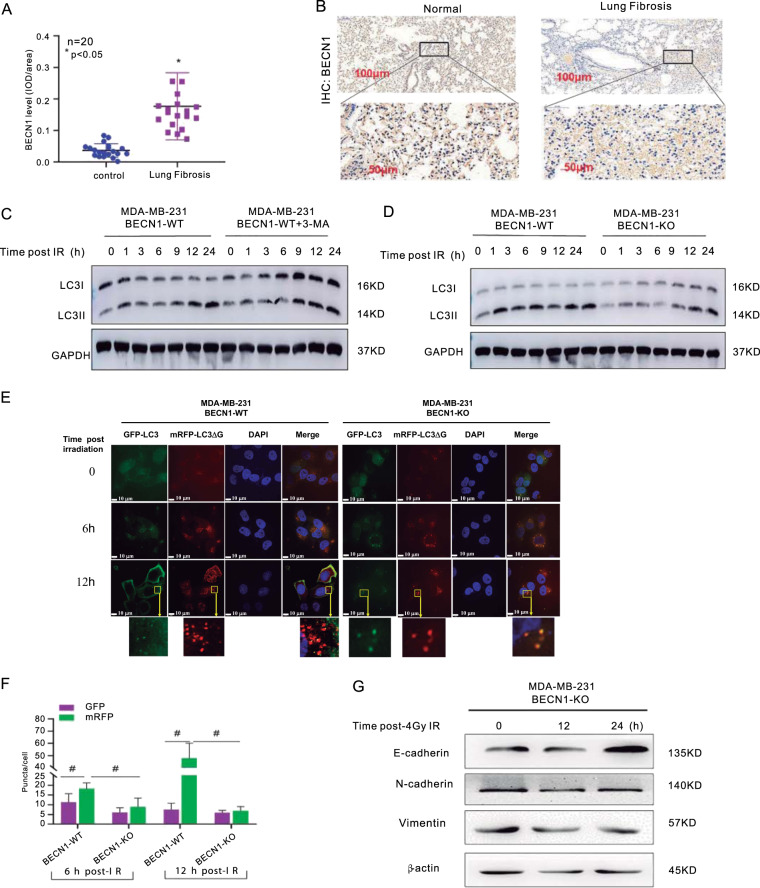
Involvement of autophagy in the regulation of IR-induced EMT. **a** The BECN1 expression level was analyzed in 20 paired mice lung fibrosis tissues with 20 Gy radiation and their normal tissues without 20 Gy radiation by calculating the integrated IOD/area using Image-Pro Plus version 6.0. Three independent experiments were performed, the student’s *t*-test was utilized to determine the p-value and the asterisk (*) indicates a significant change compared with control group. **b** IHC staining was performed to evaluate BECN1 expression in 20 paired mice lung tissues with 20 Gy radiation and their normal tissues without 20 Gy radiation. The IHC images were captured using the AxioVision Rel.4.6 computerized image system. **c** An autophagy flux experiment was performed to test the autophagy process. The protein levels of BECN1 and LC3B in MDA-MB-231 cells were detected by western blotting analysis. GAPDH was used as the control. MDA-MB-231 cells cotreated with or without 3-MA were harvested at the indicated timepoints after 4-Gy γ-ray-irradiation. **d** An autophagy flux experiment was performed to test the autophagy process. The protein levels of BECN1 and LC3B in MDA-MB-231 cells were detected by western blotting analysis. GAPDH was used as the control. MDA-MB-231 cells cotreated with or without 3-MA were harvested at the indicated timepoints after4-Gy γ-ray-irradiation. **e** The effect of BECN1 knockout on autophagy flux was examined. The cells were transfected with tandem GFP-LC3-mRFP-LC3ΔG plasmids mediated by the adenovirus, and fluorescence laser confocal microscopy was performed(bars: ×20 magnification). **f** Quantitative measurement of GFP and mRFP. The data are presented as the means ± SDs from three independent experiments; *p < 0.05 compared with the WT cells. **g** Western blot assay was performed to detect the radiation-induced EMT biomarkers’ alteration post 4 Gy radiation in MDA-MB-231 cells at indicated timepoints.

We performed immunofluorescence detection of the autophagy-related markers LC3 and WIPI2 (WD-repeat domain, phosphoinositide interacting 2), which is a member of the ATG18/WIPIs (WD-repeat protein interacting with phosphoinositide) family^14^. As shown in Fig. 1a, b, c, upregulation of BECN1 was unexpectedly observed in these cell lines, revealing the potential association of autophagy with radiation-related development of lung fibrosis. After 4 Gy irradiation, the protein levels of LC3-II were significantly increased overtime after IR, whereas the SQSTM1/P62 level was decreased; this trend was largely reversed to a certain extent by 3-MA treatment (Fig. 1d). The numbers of LC3 puncta per cells increased postradiation, but this effect was inhibited by treatment with 3-MA and wortamannin (Fig. 1e). As shown in Fig. 1f, g, the numbers of LC3 and WIPI2 puncta/dots increased postradiation, but this effect was inhibited by treatment with 3-MA. The level of BECN1 in mice radiation-induced lung fibrosis tissues are remarkably upregulated compared with the control group without radiation treatment (Fig. 2a). IHC staining was conducted and the BECN1 expression was verified to be upregulated in ice radiation-induced lung fibrosis tissues compared with the control group without radiation treatment (Fig. 2b). Collectively, these above data strongly demonstrate that autophagy and BECN1 protein expression are elevated in human cells in vitro, and mouse lung fibrosis tissues in vivo.

### BECN1 and its mediated autophagy were essential for cell response to radiation and lung fibrosis

At the early stage (0–12 h) after irradiation, a series of survival-promoting DDRs, such as cell-cycle checkpoints and DNA repair, are activated and encourage cells to recover from radiation damage. Therefore, we designed experiments in which the cells were treated with the autophagy inhibitor 3-MA at the early (0–12 h post-IR) or later stages (12–24 h post-IR). Supplementary Fig. 1a suggest that autophagy might play a protective role during the early stage (0–12 h) but not at the later stage of the response to IR. After 2- or 4-Gy irradiation, the survival rates of 3-MA-treated HeLa cells were significantly decreased compared with those of the untreated cells (Supplementary Fig. 1b). As shown in Supplementary Fig. 1c, d, the apoptosis level was markedly increased in the group treated with 3-MA at 0 to 12 h post 4-Gy-irradiation (4 Gy + 3-MA@0 h) compared with the group that was only irradiated with 4 Gy (4 Gy). These results further suggest that autophagy plays a dual-effect role in the early response of cells to irradiation, which at the early stage (0–12 h) of radiation exposure, autophagy is subjected to protective effect but at the later stage (12–48 h), it is subjected to inhibition effect.

The LC3-II level increased with increasing time post-IR, but a notably lower increase was observed in the cells cotreated with 3-MA (Fig. 2c). Autophagic flux was found to be active in irradiated BECN1-WT cells. BECN1 deficiency resulted in a significantly decreased LC3B II/I ratio in BEC1-KO cells compared with BECN1-WT cells (Fig. 2d). We used cells transfected with the tandem GFP-LC3-mRFP-LC3ΔG plasmid to further confirm the effect of BECN1-KO on autophagic flux. This fusion protein can be cleaved by the endogenous ATG4 family protease to generate equal amounts of GFP-LC3 and mRFP-LC3^15,16^. In the acidic environment of the lysosome, mRFP-LC3 (red) is markedly more stable than GFP-LC3 (green). MRFP red puncta represent the maturation of autophagolysosomes, and yellow puncta (indicating the expression of both GFP and mRFP) represent the formation of autophagosomes. A lower ratio of GFP-LC3 to RFP-LC3 puncta reflects autophagic flux. Both yellow and red puncta were observed in the cells, and a decreased ratio of GFP-LC3 to RFP-LC3 puncta was apparent following 4-Gy irradiation (Fig. 2e). However, fewer LC3 puncta and a higher ratio of GFP-LC3 to RFP-LC3 puncta were observed in the irradiated BECN1-KO cells, particularly at 12 h, compared with the BECN1-WT cells (Fig. 2e, f). As shown in Fig. 2g, at indicated timepoints after 4 Gy IR treatment, the E-cadherin expression level was increased and E-cadherin and Vimentin expression levels were decreased at the BECN1 deficiency status. These data reveal that BECN1-mediated autophagy is at least one of key events for BECN1 promotion of radiation-induced EMT.

### G_2_/M checkpoint was regulated by BECN1 in response to IR

**Fig. 3:**
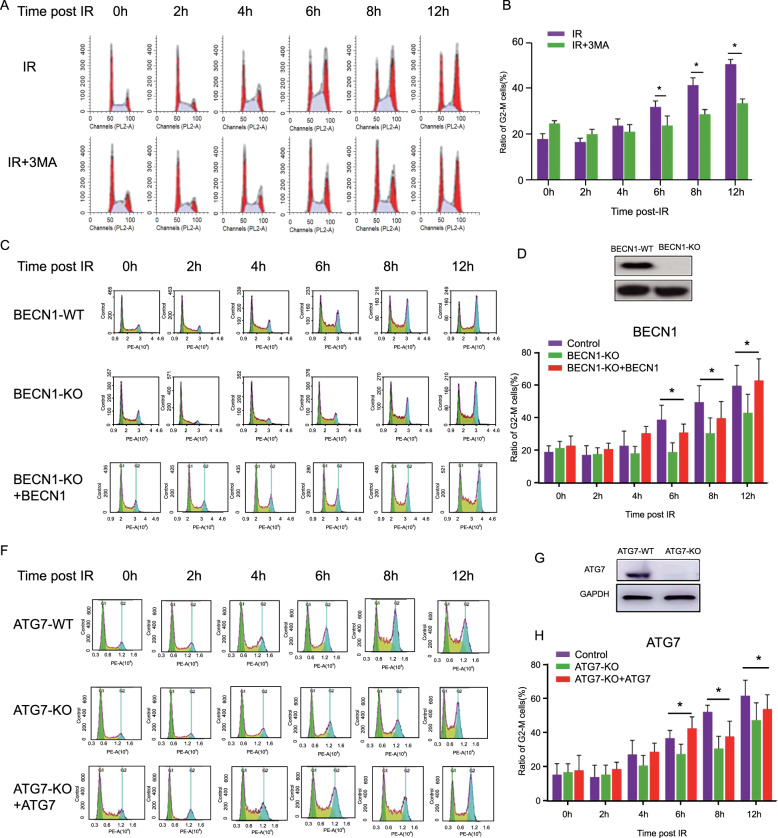
Involvement of autophagy in the regulation of IR-induced cell-cycle progression arrest. **a** Representative flow cytometry histogram of cell-cycle progression in the population of 4-Gy γ-ray-irradiated HeLa cells with or without cotreatment with 3-MA. **b** Effect of 3-MA on the G_2_ and M phase distribution in the population of irradiated cells. The data are presented as the means ± SDs from three independent experiments; *p < 0.05 compared with the control group. **c** Representative flow cytometry histogram of cell-cycle progression in the population of 4-Gy γ-ray-irradiated BECN1-WT and BECN1-KO MDA-MB-231 cells. **d** Western blotting analysis of BECN1 in BECN1-WT MDA-MB-231 cells (control) and BECN1-KO cells generated by CRISPR/Cas9. GAPDH served as the internal loading control. **e** Quantitative measurement of the proportions of 4-Gy γ-ray-irradiated BECN1-KO and control cells in the G_2_ and M phases at the indicated times after IR. The data are presented as the means ± SDs from three independent experiments; *p < 0.05 compared with the control group. **f** Representative flow cytometry histogram of cell-cycle progression in the population of 4-Gy γ-ray-irradiated ATG7-WT and AG7-KO MDA-MB-231 cells. **g** Western blotting analysis of ATG7 in ATG7-WT MDA-MB-231 cells (control) and ATG7-KO cells generated by CRISPR/Cas9. GAPDH served as the internal loading control. **h** Quantitative measurement of the proportion of 4-Gy γ-ray-irradiated ATG7-KO and control cells in the G_2_ and M phases at the indicated times after IR. The data are presented as the means ± SDs from three independent experiments; *p < 0.05 compared with the control group.

As shown in Fig. 3a, b, the percentages of G_2_- and M-phase cells increased in a time-dependent manner after 6-Gy irradiation, which indicated the induction of G_2_/M arrest and/or mitotic arrest. The cells pretreated with 3-MA exhibited relatively lower percentages of cells at the G_2_ and M phases compared with those found for the cells that were only irradiated alone (Fig. 3b). A similar result was also observed after pretreatment with chloroquine, another autophagy inhibitor (Supplementary Fig. 2). Because autophagy might participate in IR-induced cell-cycle arrest, we hypothesized that BECN1, a key regulator of autophagy, might play a critical role in the crosstalk between autophagy and cell-cycle progression. CRISPR/Cas9-mediated *BECN1*-knockout MDA-MB-231 cells (BECN1-KO) were used (Fig. 3d). As shown in Fig. 3c, e, BECN1 deficiency resulted in decreased G_2_/M arrest in response to IR compared with wild-type (WT) BECN1. Rescue experiments further showed that the restoration of BECN1 might increase G_2_/M arrest in response to IR. The silencing of BECN1 expression by specific siRNAs (Supplementary Fig. 3a) further demonstrated that autophagy-related BECN1 is involved in the regulation of irradiation-induced G_2_/M arrest (Supplementary Fig. 3b, c).

CRISPR/Cas9-mediated *ATG7*-knockout MDA-MB-231 cells were also used to further investigate the crosstalk between autophagy and cell-cycle progression, and the knockout of ATG7 expression was detected by western blotting (Fig. 3g). As shown in Fig. 3f, h, ATG7 deficiency resulted in decreased G_2_/M arrest in response to IR, which was similar to the effect observed with BECN1 deficiency.

**Fig. 4:**
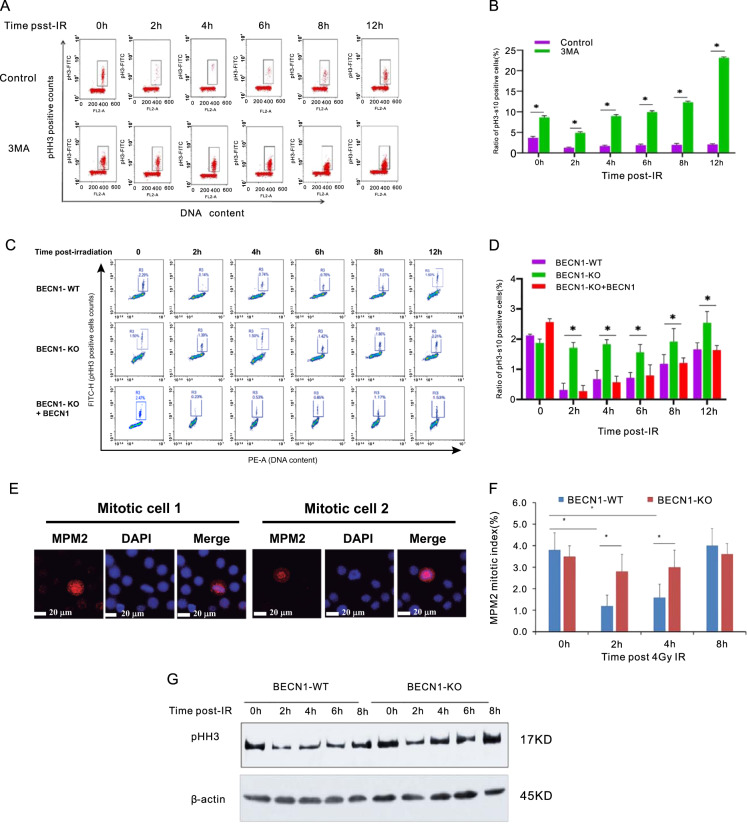
Autophagy-related BECN1 deficiency inactivates the G_2_/M checkpoint in response to IR. **a** Representative flow cytometry histograms of phosphorylated histone H3 (pHH3)-positive mitotic cells in the population of γ-ray-irradiated HeLa cells with or without 3-MA cotreatment. The cells were immunostained with pHH3(Ser10) antibody to detect the proportion of pHH3-positive mitotic cells at the indicated times after IR. **b** Quantitative measurement of pHH3(Ser10)-positive cells. The data are presented as the means ± SDs from three independent experiments; *p < 0.01 compared with the control group. **c** Representative flow cytometry histogram of the population of γ-ray-irradiated BECN1-WTor BECN1-KO MDA-MB-231 cells. The cells were immunostained with pHH3(Ser10) antibody to detect the proportion of pHH3-positive cells at the indicated times after IR. A rescue experiment was conducted by transfecting BECN1-KO MDA-MB-231 cells with lentiviral vectors expressing BECN1. **d** Quantitative measurement of pHH3(Ser10)-positive cells. The data are presented as the means ± SDs from three independent experiments; *p < 0.05 compared with the WT cells. **e** Representative image of mitotic protein monoclonal-2 (MPM2)-positive cells obtained through immunofluorescence staining (bars: ×20 magnification). **f** Quantification of MPM2 antibody-stained mitotic cells. The data are presented as the means ± SDs from three independent experiments; *p < 0.05 compared with the WT cells. **g** pHH3 was detected by Western blotting analysis. β-actin served as the internal control.

To further clarify the role of autophagy-related BECN1 in radiation-induced G2/M arrest, the cells were analyzed by flow cytometry following the immunostaining of phospho-histone H3/Ser10 (pHH3), a marker of cells in mitosis^17,18^. As shown in Fig. 3a, the ratio of pHH3-positive mitotic cells was sharply decreased in the population of control cells at 2–4 h postirradiation, which indicated that activation of the G_2_/M boundary checkpoint results in arrest at the G_2_/M transition. Eight hours post-IR, the mitotic cells in the population of cells treated with radiation and 3-MA was scarcely decreased (Fig. 4a, b). PHH3 immunofluorescence staining was also performed in BECN1*-*WT and BECN1-KO MDA-MB-231 cells (Fig. 4c, d). The proportion of pHH3-positive mitotic cells was sharply decreased in the population of BECN1*-*WT cells at 2–4 h postirradiation. In contrast, the proportion of mitotic cells in the population of BECN1*-*KO MDA-MB-231 cells was unaffected at the early stage (0–8 h) of the response to IR. At 12 h postirradiation, an increased proportion of mitotic BECN1-KO cells were observed, which suggested the occurrence of prolonged mitotic arrest in the population of BECN1-deficient cells. A representative image of MPM2-positive mitotic cells is shown in Fig. 4e. The MPM2 staining results further indicated that BECN1 deficiency liberated cells after IR-induced G_2_/M arrest, i.e., BECN1 deficiency resulted in inactivation of the G_2_/M checkpoint (Fig. 4e, f). Thus, the results clearly demonstrate that autophagy-associated BECN1 deficiency inactivates the G_2_/M checkpoint in response to IR.

### BECN1 deficiency disrupts the responses of G_2_/M checkpoint proteins to IR

**Fig. 5:**
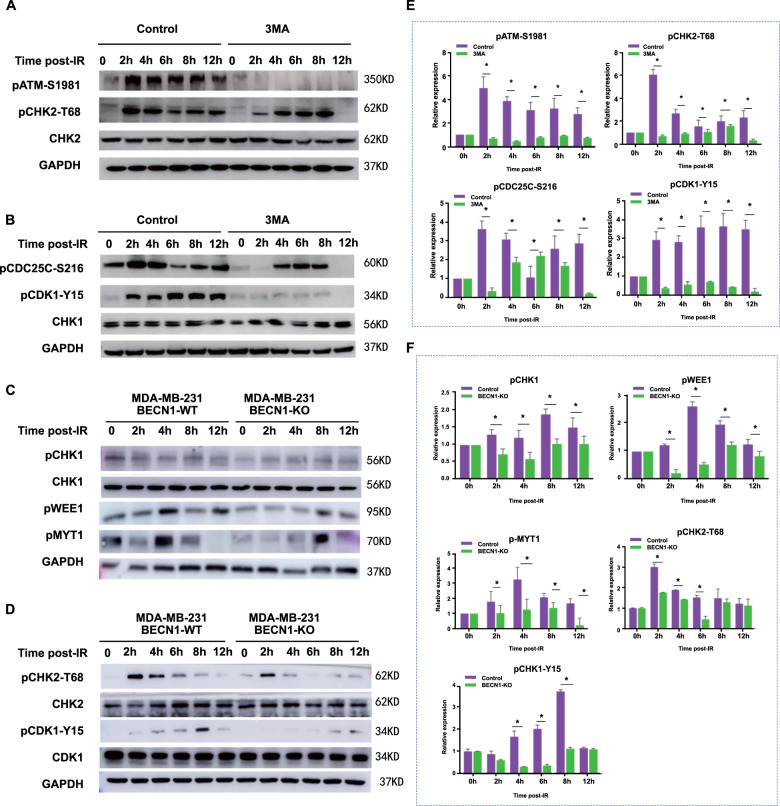
Autophagy inhibition and BECN1 deficiency disturb the response of G_2_/M checkpoint-related proteins to IR. **a** Western blotting analysis of G_2_/M checkpoint-regulating proteins (pATM, pCHK2, and CHK2) in the γ-ray-irradiated A549 cells that were cotreated or not cotreated with the autophagy inhibitor 3-MA. The proteins were detected at the indicated timepoints after IR, and GAPDH served as the internal control. **b** Western blotting analysis of the phosphorylation of G_2_/M checkpoint-regulating proteins (pCDC25C, pCDK1, and CDK1) in the γ-ray-irradiated A549 cells with or without cotreatment with the autophagy inhibitor 3-MA. The phosphorylation analysis was performed at the indicated timepoints after IR, and GAPDH served as the internal control. **c** Western blotting analysis of G_2_/M checkpoint-associated phosphorylated proteins (pCHK1, CHK1, pWEE1, and pMYT1) in the γ-ray-irradiated BECN1-WTor BECN1-KO MDA-MB-231 cells. GAPDH served as the internal control. **d** Western blotting analysis of G_2_/M checkpoint-associated phosphorylated proteins (pCHK2, CHK2, pCDK1, and CDK1) in the γ-ray-irradiated BECN1-WTor BECN1-KO MDA-MB-231 cells. GAPDH served as the internal control. **e** Quantitative measurement of the levels of pATM, pCHK2, pCDC25C, and pCDK1 in A549 cells with or without 3-MA cotreatment detected at the indicated time point after IR. The data are presented as the means ± SDs from three independent experiments; *p < 0.05 between different groups. **f** Quantitative measurement of phosphorylated protein levels of pCHK1, pWEE1, pMYT1, and pCDK1 in BECN1-WT and BECN1-KO MDA-MB-231 cells at the indicated timepoints after IR. The data are presented as the means ± SDs from three independent experiments; *p < 0.05 between different groups.

As shown in Fig. 5a, b, irradiated HeLa cells cotreated with 3-MA exhibited decreased levels of pATM, pCHK2, pCDC25C, and pCDK1 at the indicated timepoints postirradiation compared with the control cells. CDK1 dephosphorylation and CCNB1 expression are required for activation of the CDK1-CCNB1 complex^19^. This complex is maintained in an inactive state through phosphorylation of a conserved residue, tyrosine 15 (CDK1-pY15)^20^. As shown in Supplementary Fig. 4a, b, the proportion of pHH3-positive mitotic cells was sharply decreased in the population of WT cells at 2–4 h postirradiation, whereas the numbers of mitotic cells in the populationofATG7*-*KO MDA-MB-231 cells was unaffected. ATG7 deficiency resulted in decreased G_2_/M arrest in response to IR. A rescue experiment showed that restoration of the expression of ATG7 could liberate cells from G_2_/M arrest in response to IR. Compared with WT cells, a notably lower increase in LC3-II and slower degradation of p62 protein were observedATG7 KO cells with increasing irradiation time (Supplementary Fig. 4c).

The levels of phosphorylated pCHK1, pCHK2, pWEE1, and pCDK1 were increased in BECN1-WT MDA-MB-231 cells after 6-Gy irradiation, whereas no obvious change or only a weak increase in the levels of nonphosphorylated CHK2, WEE1, and CDK1 were detected in BECN1-WT MDA-MB-231 cells. Overall, the expression of MYT1 in BECN1-KO cells was lower than that in WT cells at all examined times postirradiation (Supplementary Fig. 5a, b). The changes in the phosphorylation levels of the related proteins are shown in Fig. 5c, d. Lower levels of pCHK1, pCHK2, pWEE1, pMYT1, and pCDK1 were observed in BECN1-KO MDA-MB-231 cells compared with BECN1-WT cells at the indicated timepoints postirradiation. These results suggest that (i) CDK1 is phosphorylated and inactivated in WT cells following irradiation but is maintained in a dephosphorylated state in BECN1-KO cells; (ii) BECN1 deficiency might promote the dephosphorylation of CDK1; and (iii) Inactivation of the G_2_/M checkpoint due to autophagy inhibition or BECN1 deficiency might be partially due to dysregulation of the ATM/CHK2/CDC25C/CDK1 signaling pathway.

### BECN1 deficiency disrupts the dissociation of CDC25C from CDK1 following irradiation

**Fig. 6:**
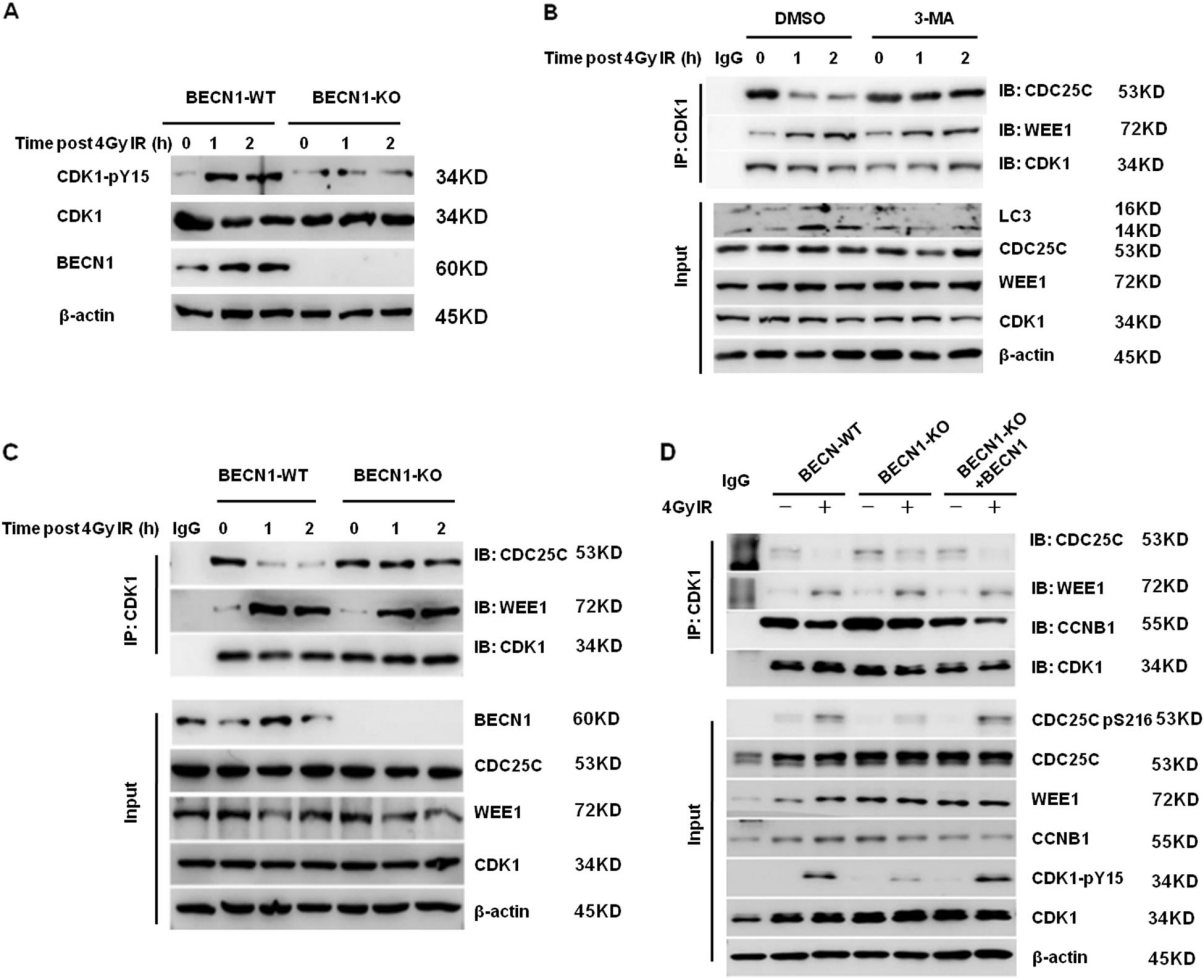
BECN1 deficiency and autophagy inhibition inhibits the dissociation of CDC25C from the CDK1 complex after irradiation. **a** The levels of phosphorylated CDK1-pY15 protein in BECN1-KO and BECN1-WT MDA-MB-231 cells were detected 1 and 2 h after 4-Gy irradiation by western blot analysis. β-actin served as the internal loading control. **b** Effects of 3-MA treatment on the protein–protein interactions of the CDK1 complex in MDA-MB-231 cells after irradiation. Cell lysates were collected from DMSO-treated MDA-MB-231 cells and 3-MA-treated cells at 2 h after 4-Gy irradiation, and immunoprecipitates were prepared with anti-CDK1 or anti-IgG antibodies. Western blot analysis was performed using anti-CDK1, anti-CDC25C, and anti-WEEl antibodies. β-actin was used as the internal loading control. **c** Effects of BECN1 deficiency on the protein–protein interactions of the CDK1 complex after irradiation. Cell lysates were collected from BECN1-WT and BECN1-KO MDA-MB-231 cells at the indicated timepoints after 4-Gy irradiation, and immunoprecipitates were prepared with anti-CDK1 or anti-IgG antibodies. Western blotting analysis was performed using anti-CDK1, anti-CDC25C, and anti-WEEl antibodies. β-actin was used as the internal loading control. **d** Effects of BECN1 deficiency and overexpression on the protein–protein interactions of the CDK1 complex in MDA-MB-231 cells with or without 4-Gy irradiation. Cell lysates were collected from BECN1-WT cells, BECN1-KO cells, and BECN1-KO cells transfected with exogenous BECN1 mediated by lentiviral vectors with or without 4-Gy irradiation, and immunoprecipitates were prepared with anti-CDK1 or anti-IgG antibodies. Western blotting analysis was performed using the indicated antibodies. β-actin was used as the internal loading control.

As shown in Fig. 6a, the phosphorylated CDK1-pY15 levels postirradiation were increased in the BECN1-WT cells, and this effect is attributed to inactivation of the phosphatase CDC25C^21^. However, the expression of CDK1-pY15 in BECN1-KO cells was barely changed after irradiation, which further indicated that CDK1 dephosphorylation in response to radiation is associated with BECN1. As shown in Fig. 5b, the interaction between CDK1 and CDC25C was sharply decreased in WT cells following irradiation, which suggested the dissociation of these two proteins. However, the cotreatment of the cells with the autophagy inhibitor 3-MA abrogated the radiation-induced dissociation of CDC25C from CDK1 (Fig. 6b). The interaction of CDK1 with CDC25C was detected in both BECN1-KO and BECN1-WT cells under normal growth conditions. CDC25C rapidly dissociated from CDK1 in BECN1-WT cells within 1 and 2 h after 4-Gy irradiation, whereas the interaction of CDC25C with CDK1 was not disrupted in the irradiated BECN1-KO cells (Fig. 6c), which is consistent with the effect of 3-MA treatment (Fig. 5b). Importantly, the restoration of BECN1 expression in BECN1-KO cells through adenovirus-mediated transfection of a BECN1-expressing vector resulted in the irradiation-induced dissociation of CDC25C from CDK1 (Fig. 6d). In addition, CDK1 CoIP results indicated that the interaction between CDK1 and WEE1 increased after 4-Gy irradiation in both BECN1-KO and BECN1-WT cells, which indicated that BECN1 does not influence the interaction between CKD1 and WEE1 (Fig. 6c, d).

**Fig. 7:**
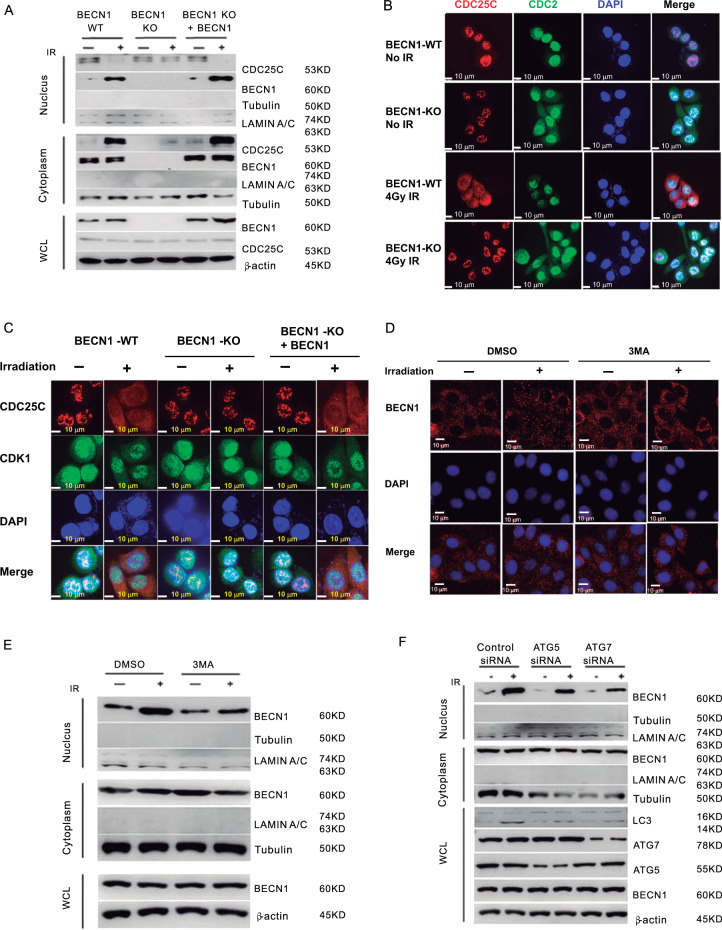
The radiation-induced cytoplasmic translocation of CDC25C is blocked under BECN1-deficient conditions. **a** The levels of CDC25C and BECN1 proteins in the cytoplasm and nucleus, respectively, in BECN1-KO and BECN1-WT MDA-MB-231 cells after 4-Gy irradiation were detected by Western blot analysis. β-actin served as the internal loading control. Tubulin and LAMIN A/C proteins were detected as the control cytoplasmic and nuclear proteins, respectively. Exogenous BECN1-expressing vectors were transfected into BECN1-KO cells for the rescue experiment. **b** CDC25C and CDK1 in BECN1-WT and BECN1-KO MDA-MB-231 cells with or without 4-Gy γ-ray-irradiation were detected by immunofluorescence confocal microscopy(bars: ×20 magnification). **c** CDC25C and CDK1 in BECN1-WT and BECN1-KO MDA-MB-231 cells with or without 4-Gy γ-ray-irradiation were detected by immunofluorescence confocal microscopy. Exogenous BECN1-expressing vectors were transfected into BECN1-KO cells for the rescue experiment(bars: ×20 magnification). **d**. The BECN1 levels after 4-Gy γ-ray-irradiation with or without 3-MA treatment were assayed by immunofluorescence microscopy (bars: ×20 magnification). **e** The levels of BECN1, tubulin and LAMIN A/C proteins in the cytoplasm and nucleusafter 4-Gy irradiation with or without cotreatment with 3-MA were detected by Western blot analysis. β-actin served as the internal loading control. **f** Effects of siRNA-mediated knockdown of ATG5 and ATG7 on the radiation-induced nuclear translocation of BECN1.

As shown in Fig. 7a, increased levels of BECN1 in the nucleus and CDC25C in the cytoplasm were detected in BECN1-WT cells following irradiation. However, in BECN1-KO cells, the radiation-induced cytoplasmic translocation of CDC25C was largely blocked. Immunofluorescence staining also demonstrated that the cytoplasmic translocation of CDC25C was blocked in irradiated BECN1-KO cells and could be restored following transfection of an exogenous BECN1-expressing vector (Fig. 7b, c). These results are consistent with the effects of BECN1 on the interaction between CDC25C and CDK1 and the dephosphorylation of CDK1 (Fig. 6). The immunofluorescence staining assay results clearly showed that the radiation-induced nuclear translocation of BECN1 was largely attenuated by 3-MA (Fig. 7d). In addition, a western blot analysis indicated that irradiation increased the nuclear translocation of BECN1, and this effect was reduced by treatment with 3-MA (Fig. 7e). Moreover, the suppression of ATG5 and ATG7 expression by specific siRNAs inhibited radiation-induced autophagy, also prevented the radiation-induced nuclear translocation of BECN1 (Fig. 7f). These data demonstrate the following: (i) CDK1 interacts with WEE1 and CDC25C to form multi-protein complexes in BECN1-expressing MDA-MB-231 cells. (ii) BECN1 deficiency inhibition prevents the translocation of CDC25C from the nucleus to the cytoplasm. (iii) Consequently, CDK1 is found in a state of dephosphorylation in BECN1-deficient cells, leading to inactivation of the G_2_/M checkpoint and cell-cycle progression from the G_2_ to the M phase without arrest, even under the stress induced by radiation injury.

**Fig. 8:**
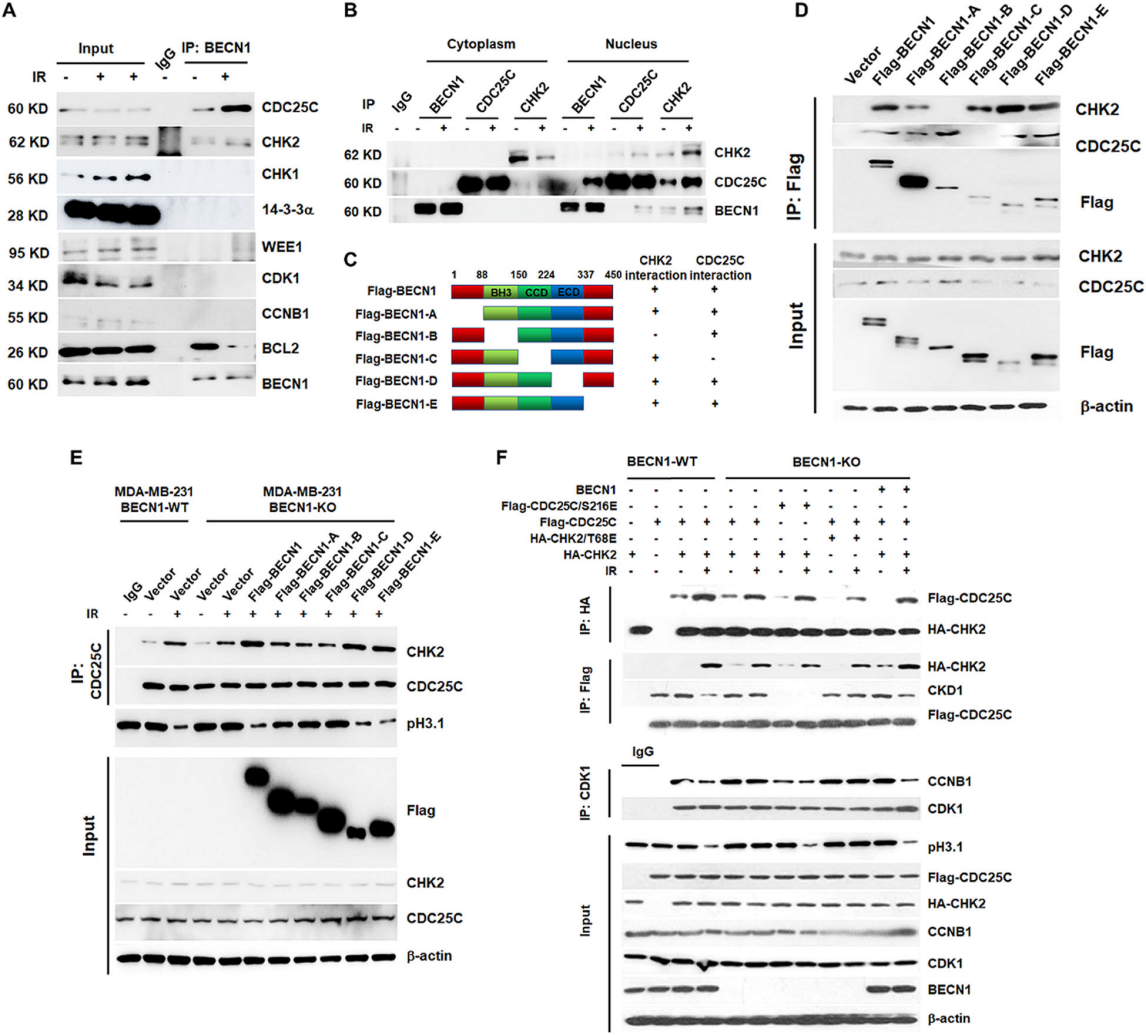
BECN1 mediates the interaction of CDC25C with CHK2. **a** The interactions of BECN1 with CDC25C, CHK2, CHK1, 13-3-3α, WEE1, CDK1, CCNB1, and BCL2in MDA-MB-231 cells with or without 4-Gy irradiation were assayed, and immunoprecipitates were prepared with anti-BECN1 or anti-IgG antibodies. Western blot analyses were performed using the indicated antibodies. **b** The interactions of BECN1 with CDC25C and CHK2 in either the cytoplasm or nucleusof MDA-MB-231 cells with or without 4-Gy irradiation were assayed, and immunoprecipitates were prepared with anti-BECN1, anti-CHK2, anti-CDC25C, or anti-IgG antibodies. Western blot analysis was performed using anti-CDC25C and anti-CHK2 antibodies. **c***BECN1*-truncated mutants were constructed according to the listed illustrator. **d** Flag-BECN1, flag-BECN1-A, flag-BECN1-B, flag-BECN1-C, flag-BECN1-D, flag-BECN1-E, and empty vector were overexpressed in MDA-MB-231 cells. Immunoprecipitation was performed using a flag antibody. **e** Flag-BECN1, flag-BECN1-A, flag-BECN1-B, flag-BECN1-C, flag-BECN1-D, flag-BECN1-E, and empty vector were overexpressed in BECN1-KO MDA-MB-231 cells. Immunoprecipitation was performed using anti-CDC25 antibody, and western blot analysis was performed using anti-CDC25C and anti-CHK2 antibodies. **f** The cells were transfected with the indicated Flag- or HA-tagged expression vectors, and the immunoprecipitations were performed using anti-HA, anti-Flag, or anti-IgG antibodies. Western blot analyses were performed using anti-Flag, anti-HA, anti-CDK1, and anti-CCNB1 antibodies. β-actin served as the internal loading control.

We then wondered whether the dissociation of CDC25C from CDK1 and its translocation from the nucleus to cytoplasm are directly mediated by BECN1. As shown in Fig. 8a, there exists interactions among BECN1, CDC25C, and CHK2, and these interactions increased following irradiation. We also observed an interaction between BECN1 and BCL2, and this interaction was weakened after irradiation. We did not detect any interactions between BECN1 and CHK1 or among CDK1, CCNB1, and WEE1 (Fig. 8a). As shown in Fig. 8b, the interaction of BECN1 with CDC25C and CHK2 only occurred in the nucleus, and this interaction increased following irradiation. To determine the domain(s) of BECN1 that mediate its interactions with CDC25C and CHK2, we generated a series of truncated BECN1 mutants (Fig. 8c). As shown in Fig. 8d, the BECN1-B mutant, in which amino acids 89–155 were deleted, was no longer able to interact with CHK2, and the BECN1-C mutant, in which amino acids 151–224 were deleted, was no longer able to interact with CDC25C.

As shown in Fig. 8e, the interaction between CDC25C and CHK2 was strengthened in BECN1-WT cells following irradiation. However, in BECN1-KO cells, the radiation-enhanced interaction between CDC25C and CHK2 was only observed after transfection of a vector directing the expression of full-length BECN1 or the BECN1-D or BECN1-E mutants. The BECN1-B and BECN1-C mutants did not interact with CHK2 or CDC25C. These results indicate that the interaction/phosphorylation of CDC25C with/by CHK2 in response to IR is mediated by BECN1.

Finally, we investigated the effects of BECN1 on the assembly/interaction of the G_2_/M checkpoint complex CHK2/CDC25C/CDK1/CCNB1 and its association with CHK2 kinase activity and the phosphorylation of CDC25C/S216 in response to IR (Fig. 8f). An HA-tagged CHK2 immunoprecipitation (IP:HA) assay showed that the interaction between exogenous HA-CHK2 and Flag-CDC25C BECN1-WT cells increased following irradiation. The interaction between HA-CHK2 and Flag-CDC25C in BECN1-KO cells was lower than that in WT cells, regardless of irradiation. However, restoration of the expression of BECN1 allowed the radiation-induced interaction of CHK2 and CDC25C in BECN1-KO cells.

A Flag-tagged CDC25C immunoprecipitation assay (IP: Flag) showed that the interaction between exogenous HA-tagged CHK2 and Flag-tagged CDC25C in BECN1-WT cells was also enhanced following IR, whereas the interaction of Flag-tagged CDC25C with endogenous CDK1 in these cells decreased following irradiation (Fig. 8f). Similarly, in BECN1-KO cells, irradiation weakened the interaction of Flag-CDC25C with HA-CHK2 but did not decrease the interaction of Flag-CDC25C with CDK1. The phosphorylation-mimic mutant CDC25/S216E was no longer able to interact with CDK1. The restoration of BECN1 expression increased the interaction of CDC25C with CHK2 in BECN1-KO cells and decreased the interaction of CDC25C with CDK1 to a level similar to that observed in BECN1-WT cells in response to radiation. A high level of interaction was found between CDK1 and CCNB1 in BECN1-KO cells, and this level decreased following transfection of the phosphorylation-mimic CDC25C-S216E expression vector. Restoration of BECN1 expression decreased the interaction of CDK1 with CCNB1 in irradiated BECN1-KO cells.

### The BECN1 and CDK1 expression levels are increased in breast cancer tissue samples

To determine whether the expression of *BECN1* and *CDK1* are altered in breast cancer tissues, gene expression data from the Gene Expression Omnibus (GEO) database (accession numbers GSE81838 and GSE65194) and the breast cancer patient dataset from the Cancer Genome Atlas (TCGA) were analyzed^22^. As shown in Supplementary Fig. 6a, 93 genes overlapped among the three datasetsGSE65194, GSE81838, and TCGA datasets, of which BECN1 and CDK1 were both upregulated in breast cancer tissue compared with normal tissue. Supplementary Fig. 6b presents the relative expression levels of several essential autophagy-related genes, including *BECN1* and G_2_/M-regulated genes, such as *CDK1*, *CDC25C*, and *CHK1*, in breast cancer and normal tissues in the TCGA dataset. We also found that both *BECN1* and *CDK1* are upregulated in breast cancer tissue compared with normal tissue (Supplementary Fig. 6c). Several essential autophagy-related and G2/M-regulating genes, including *BECN1*, *CDK1*, and *CDC25C*, are coexpressed; in particular, *CDK1* is associated with both autophagy-related and G2/M-regulating genes (Supplementary Fig. 6d).

Therefore, BECN1 was translocated into the nucleus following IR, where it mediated the interaction of CDC25C with CHK2, prompted the phosphorylation of CDC25C and its dissociation from CDK1 and thus resulted in the inactivation of the CDK1/CCNB1 complex and arrest at the G_2_/M transition in the cell cycle, leading the CDK1 overexpression to promote the radiation-induced EMT (Supplementary Fig. 7).

## Discussion

Autophagy and cell-cycle arrest are two critical cellular responses to IR, and autophagy is induced even as part of the radiation-induced bystander effect^23,24^. Because initiation is potentiated by the impairment of autophagy through the disruption of core autophagy genes and autophagy-defective tumor cells also display a dysregulated cell cycle^25^, we, in contrast to previous studies, used the autophagy inhibitor 3-MA and BECN1-KO cancer cells to directly determine the role of autophagy in G_2_/M arrest. The results of our study suggest that BECN1 deficiency enhances cellular sensitivity to IR, induces escape from the G_2_/M checkpoint after irradiation and promotes the G_2_/M transition without arrest. These two events [(1) the suppression of autophagy post-IR promotes cell death and suppresses proliferation and (2) the suppression of autophagy induces escape from the G_2_/M checkpoint and promotes the G_2_/M transition] appear to be but are not actually contradictory. On the one hand, the inhibition of autophagy can promote the G_2_/M transition in unrepaired cells, and on the other hand, mitotic arrest can be induced in cells damaged by radiation. Moreover, the cells that escape G_2_/M arrest enter the M phase without undergoing adequate repair, which will likely result in mitotic catastrophic cell death^26^.

BECN1 is a key protein in the regulation of autophagy through the activation of VPS34^27^. Xiao et al. demonstrated that macroautophagy is regulated by the cell-cycle protein Sdk1, which impairs the interaction of BECN1 with VPS34^28^. CDK1 is an important player in macroautophagy suppression during the M phase. CDK1 can directly phosphorylate VPS34, which prevents formation of the BECN1-VPS34 complex and leads to decreased autophagy in M-phase cells^29^. In contrast, CDK inhibitors stimulate autophagy by releasing BECN1, which results in the promotion of tumor growth^30^. Our study revealed the involvement of autophagy in the regulation of the G_2_/M checkpoint. Autophagy dysregulation can disrupt arrest at the G_2_/M transition following irradiation, primarily by affecting the dissociation of CDC25C from CDK1 and decreasing the phosphorylation of ATM, CHK1, CHK2, and CDK1 in the absence of BECN1. To the best of our knowledge, this study provides the first demonstration that autophagy and the G_2_/M transition are associated with the dephosphorylation or phosphorylation of various G_2_/M-regulating proteins.

Progression from the G_2_ to the M phase is driven by activation of the CDK1/CCNB1 complex^31^. Our study provides further evidence clarifying the mechanism through which autophagy regulates the G_2_/M transition. As indicated by previous studies, basal autophagy occurs at different phases of the cell cycle in response to environmental genotoxins such as IR^32–34^. Li et al. demonstrated that increasing the expression of ATG5 inATG5-deficient cells rescues the cells from G_2_/M arrest, whereas expression of the ATG5-K130R mutant does not produce this effect; the rescue from G_2_/M arrest coincides with increased levels of pCDK1 and CDKN1A/p21^35^. Jia et al. demonstrated that autophagy regulates the negative cell-cycle regulator CDKN1B in naïve T cells and reduces its abundance in T cells after their activation to enter the cell cycle^36^. Liu et al. reported that MJ-66, a tumor growth inhibitor, induces glioma cell-cycle arrest at the G_2_/M transition and increases the expression of PDK1 and CDK1^31^. In the present study, we used CoIP assays to reveal that in BECN1-WT cells, CDC25C dissociates from the CDK1 complex after irradiation, which results in increased phosphorylation of CDK1 by WEE1 and thus arrest at the G_2_/M transition in the cell cycle. However, in BECN1-deficient cells, the dissociation of CDC25C from CDK1 is attenuated, which results in CDK1 dephosphorylation and promotion of the G_2_/M transition even under the stress induced by radiation injury. Xu et al. reported that following B19V infection, activated ATR phosphorylates CDC25C, which in turn inactivates the CCNB1-CDK1 complex^37^. Jaceosidin, isolated from Japanese mugwort, induces arrest at the G_2_/M transition in the cell cycle through inactivation of the CDC25C-CDK1 complex^38^. Vera et al. reported that both CDK1 and WEE1 are mediators of G_2_/M arrest^39^. Our study showed that BECN1 interacts with CDC25C and CHK2 using different domains. BECN1 deficiency decreased the interaction of CDC25C with CHK2 and stabilized the interaction of CDC25C with CDK1 even in the presence of IR. Our study elucidated that under IR stress, autophagy promotes G_2_/M arrest by targeting the formation of the CDC25C-CHK2, CDK1-CDC25C, and CDK1-WEE1 complexes.

In conclusion, our study demonstrates that BECN1 plays a role in promotion of radiation-induced G2/M arrest (Figs. 7g and 8). However, whether such functions of BECN1 in G2/M arrest is dependent or independent on its autophagy-related roles is necessary to further identify.

## Materials and methods

### Reagents and antibodies

The reagents used in this study were as follows: liposomes were purchased from Invitrogen (Carlsbad, CA, USA); glycine, lauryl sodium sulfate, tetramethylethylenediamine, TRIzol, and tris(hydroxymethyl)aminomethane were purchased from Amresco (Solon, OH, USA); acrylic amide was purchased from Merck (Darmstadt, Germany); bovine serum albumin (BSA) was purchased from Roche (Basel, Switzerland); fluorescent protein solutions were purchased from Pierce (Rockford, IL, USA); ammonium peroxydisulfate, dimethyl sulfoxide (DMSO), *N*, *N’*-methylenebisacrylamide, and puromycin were purchased from Sigma (St. Louis, MO, USA); trypsin, Dulbecco’s modified Eagle’s medium (DMEM) with high glucose, and fetal bovine serum were purchased from HyClone (Logan, UT, USA); PrimeSTAR DNA polymerase and T4 DNA ligase were purchased from TaKaRa (Tokyo, Japan); GAPDH, P62, CHK1, CHK2, MYTl, WEEl, CDC25, CDC25C, ATM, CDK1, LC3, p68-CHK1, p216-CDC25C, p15-CDK1, p1981-ATM, SQSTM1/P62, pCHK2, pCHK1, CDC, pCDC25C, and MPM2 antibodies were purchased from Santa Cruz Biotechnology (Dallas, TX, USA), Cell Signaling Technology (Danvers, MA, USA), or Millipore/Upstate(NY, USA); and the pLKO.1 plasmid was purchased from Sigma (Darmstadt, Germany).

### Cell lines, mouse model, and irradiation conditions

Human A549 cells and the human TNBC MDA-MB-231 cell line were purchased from the American Type Culture Collection (ATCC, Manassas, VA, USA). The cells were cultured in DMEM (a high-glucose medium containing penicillin, streptomycin, and 10% FBS) and incubated at 37 °C under 5% CO2. The BECN1-KO MDA-MB-231 cell line was successfully established previously in our laboratory using the CRISPR/Cas9 system according to the free online design tool (http://crispr.mit.edu/) 7. The cells were irradiated with ^60^Co γ-rays at a dose rate of 127.15 cGy/min at room temperature at the Institute of Radiation Medicine, Academy of Military Medical Sciences (Beijing, China). Twenty C57BL/6 male mice (6–7 week old) were divided into two groups randomly, 20 Gy γ-rays were used to induce the lung fibrosis model. Mice were anesthetized with intraperitoneal sodium pentobarbital (80 mg/kg). Lung tissues were collected for further assay. The animal study has been approved by ethics committee of Xiangya School of Public Health, Central South University.

### Cell transfection

The cells were passaged the day before transfection. After the cells were grown to 60% density, *BECN1* siRNA knockdown was conducted through the transient transfection of validated *BECN1* siRNA (sense, 5’-GCUGCCGUUAUACUGUUCUTT-3’, antisense, 5’-AGAACAGUAUAACGGCAGCTT-3’) using Lipofectamine 2000 (Thermo Fisher Scientific, Waltham, MA, USA) following the manufacturer’s instructions. Scrambled siRNA was used as the negative control. Forty-eight hours after transfection, the cells were collected for further experiments.

### Cell proliferation and colony formation assays

Cell proliferation was assessed using the CCK-8 colorimetric assay (Dojindo Molecular Technologies, Kumamoto, Japan). The cells were divided into the following treatment groups: 0 Gy, 3-MA, 4 Gy + 3-MA, 3-MA at 0 h, 4 Gy, and 4 Gy + 3-MA. The cells were then cultured in a 96-well plate at a density of 4 × 10^3^ cells/well at 37 °C in the presence of 5% CO_2_for 12, 24, 48, and 72 h, and the level of cell proliferation was then assessed. The optical density (OD) of each well at 450 nm was read using a Multiskan GO microplate reader (Thermo Fisher Scientific). Each experiment was performed in triplicate.

The colony formation ability was used to assess the cell survival percentage. After treatment with 3-MA, 2 Gy, 2 Gy + 3-MA, 4 Gy, or 4 Gy + 3-MA, the cells were seeded into 60-mm culture dishes at a density of 1000 cells/dish. After 2 weeks, the cells were stained with crystal violet. The number of microscopic colonies with more than 50 cells was counted. The cell survival ratio based on the number of colony-forming irradiated cells compared with that of the control cells was calculated.

### Western blotting analysis

A Western blotting analysis was performed for the detection of proteins or phosphorylated proteins. Briefly, the samples were treated with lysis buffer, subjected to sodium dodecyl sulfate-polyacrylamide gel electrophoresis (SDS-PAGE), and transferred to polyvinylidene fluoride membranes. After the membranes were blocked with 5% nonfat milk in Tris-buffered saline containing Tween-20 (TBST) for 2 h, the membranes were incubated with primary and secondary antibodies overnight at 4 °C. An ImageQuant LAS500 system (Molecular Dynamics, Sunnyvale, CA, USA) was used to visualize the bands. Details of the Western blot analysis can be found in our previous publications^34,40–43^.

### Apoptosis detection

In this study, apoptosis was assessed using a Fluorescein Isothiocyanate (FITC)-Annexin V Apoptosis Detection Kit (BD Pharmingen, San Diego, CA, USA) following the manufacturer’s instructions. The cells were treated with or without 3-MA, subjected to 4-Gy irradiation and harvested at 24, 48, or 72 h after irradiation. The cells were washed twice with 3 ml of phosphate-buffered saline (PBS), and RNase A was then added. The cells were centrifuged, resuspended in PBS and transferred to clean Eppendorf tubes, and 100 µl of 1× Annexin V-binding solution was then added to the cells to form a suspension of 1 × 10 cells/ml. Subsequently, 5 μl of FITC-conjugated Annexin V and propidium iodide (PI) solution (40 μg/ml PI and 0.1% Triton X-100 in PBS buffer) was added. The cells were then incubated for 15 min at room temperature in the dark, and 400 μl of 1× binding buffer was then added. The cells were then analyzed by flow cytometry^44^.

### Cell-cycle analysis and G_2_/M arrest assay

The cells were seeded into 35-mm culture dishes at a density of 70–80% per dish. The cells were treated or not treated with 3-MA, subjected to irradiation 2 h after the pretreatment, and harvested at the indicated timepoints (0, 2, 4, 6, 8, or 12 h) after irradiation. After the medium was removed, the cells were treated with RNase A (62 μg/ml) and incubated at 37 °C for 30 min. The cells were stained with PI solution, and the cell-cycle distribution was analyzed by flow cytometry.

For the comparison of G_2_/M arrest between radiation-induced, 3-MA-treated, and BECN1-knockout cells, the mitotic cells were counted using a FACSCalibur flow cytometer (BD Pharmingen). Cells that were treated or not treated with 3-MA or in which BECN1 was knocked out were subjected or not subjected to irradiation and harvested at the indicated timepoints (0, 2, 4, 6, 8, or 12 h) postirradiation. After irradiation, PBS was added to suspend the cells; the cells were then centrifuged at 2000 rpm, and 0.25% Triton X-100 was added to induce membrane rupture. After the addition of 40 μl of 1% BSA containing anti-Ser10-phosphorylated histone H3 antibody, the cells were incubated for 50 min at room temperature, and 80 μl of 1% BSA containing a FITC-tagged secondary antibody was then added. The cells were then incubated for 30 min and stained with PI solution (20 μg/ml) for 10–30 min at room temperature, and the mitosis stage was analyzed by flow cytometry. Two‐dimensional dot plots were generated using ModFit LT software (Verity Software House, Inc., Topsham, ME, USA).

### Coimmunoprecipitation (CoIP)

For the CoIP assay, normal MDA-MB-231 cells and BECN1-KO MDA-MB-231 cells that were subjected or not subjected to 4-Gy irradiation were washed, harvested, and lysed with PBS buffer containing 50 nM Tris-base, 1 mM EDTA, 1% NP-40, and 1× protease inhibitor cocktail. The lysates were centrifuged, and the supernatant was collected for the CoIP assay using the Pierce Classic IP Kit (Thermo Scientific). CDK1 antibody was used to form immunocomplexes with CDC25C and WEEl, and WEEl antibody was used to form immunocomplexes with CDK1. These immunocomplexes were isolated by 8% SDS-PAGE, washed, and eluted, and the protein interactions were detected by western blotting.

### Immunofluorescence staining and laser confocal microscopy

Autophagy and subcellular protein localization were analyzed by immunofluorescence staining and laser confocal microscopy observations. Autophagy was analyzed by quantifying the formation of puncta of the autophagy biomarker GFP-LC3 by immunofluorescence staining and laser confocal microscopy. HeLa cells were seeded in glass chamber slides. After 12 h, the cells were transfected with GFP-LC3 using Lipofectamine 2000. Four hours after transfection, the cells were irradiated with 4 Gy and treated with 0.25% Triton X-100, and the nuclei were stained with DAPI for visualization. The GFP-LC3 puncta per cell were counted. The samples were observed using an LSM 510 laser-scanning confocal microscope (Zeiss, Germany).

### Coexpression network construction

To construct a coexpression network between autophagy-mediated genes and G_2_/M checkpoint genes, we analyzed the differential expression of autophagy-mediated mRNAs and G_2_/M checkpoint mRNAs (Supplementary Table 1) using gene expression data obtained from the GEO database (Accession Nos. GSE65194 and GSE81838) (https://www.ncbi.nlm.nih.gov/) and the breast cancer dataset from the TCGA (https://cancergenome.nih.gov/). The ranks of essential autophagy genes and G_2_/M checkpoint genes were determined by the absolute differences in their expression between the control and breast cancer groups. For each pair analyzed, we used the Pearson correlation test to detect significant correlations. Only Pearson correlation coefficients ≥0.9 (*p* < 0.01) were used to construct the network and generate visual representations.

## Supplementary information

Supplementary figure1

Supplementary figure2

Supplementary figure3

Supplementary figure4

Supplementary figure5

Supplementary figure6

Supplementary figure7

## References

[1] Ye H (2016). Chloroquine, an autophagy inhibitor, potentiates the radiosensitivity of glioma initiating cells by inhibiting autophagy and activating apoptosis. BMC Neurol..

[2] Yu L (2012). DAB2IP regulates autophagy in prostate cancer in response to combined treatment of radiation and a DNA-PKcs inhibitor. Neoplasia.

[3] Kuwahara Y (2018). Association between radiation-induced cell death and clinically relevant radioresistance. Histochem. Cell Biol..

[4] Taylor MA, Das BC, Ray SK (2018). Targeting autophagy for combating chemoresistance and radioresistance in glioblastoma. Apoptosis Int. J. Program. Cell death.

[5] Chen C, Wang K, Wang Q, Wang X (2018). LncRNA HULC mediates radioresistance via autophagy in prostate cancer cells. Braz. J. Med. Biol. Res..

[6] Zou YM (2014). Hypoxia-induced autophagy contributes to radioresistance via c-Jun-mediated Beclin1 expression in lung cancer cells. J. Huazhong Univ. Sci. Technol. Med. Sci..

[7] Wu CL (2018). BECN1-knockout impairs tumor growth, migration and invasion by suppressing the cell cycle and partially suppressing the epithelial-mesenchymal transition of human triple-negative breast cancer cells. Int. J. Oncol..

[8] Li B (2017). Tumor-initiating cells contribute to radiation resistance in primary human renal clear cell carcinomas by activating the DNA damage checkpoint response. Oncol. Lett..

[9] Carruthers R (2015). Abrogation of radioresistance in glioblastoma stem-like cells by inhibition of ATM kinase. Mol. Oncol..

[10] Mei Y, Glover K, Su M, Sinha SC (2016). Conformational flexibility of BECN1: essential to its key role in autophagy and beyond. Protein Sci..

[11] Chen K (2014). Artesunate induces G2/M cell cycle arrest through autophagy induction in breast cancer cells. AntiCancer Drugs.

[12] Menon MB, Dhamija S (2018). Beclin 1 phosphorylation—at the center of autophagy regularization. Front. Cell Dev. Biol..

[13] Zhong R (2018). Deoxycytidine kinase participates in the regulation of radiation-induced autophagy and apoptosis in breast cancer cells. Int. J. Oncol..

[14] Vicinanza M, Puri C, Rubinsztein DC (2019). Coincidence detection of RAB11A and PI(3)P by WIPI2 directs autophagosome formation. Oncotarget.

[15] Morishita H, Kaizuka T, Hama Y, Mizushima N (2017). A new probe to measure autophagic flux in vitro and in vivo. Autophagy.

[16] Xu J (2018). Omi/HtrA2 participates in age-related autophagic deficiency in rat liver. Aging Dis..

[17] Moniz LS, Stambolic V (2011). Nek10 mediates G2/M cell cycle arrest and MEK autoactivation in response to UV irradiation. Mol. Cell. Biol..

[18] Huang B (2014). DNA-PKcs associates with PLK1 and is involved in proper chromosome segregation and cytokinesis. J. Cell. Biochem..

[19] Wang J (2018). Tetrandrine enhances radiosensitivity through the CDC25C/CDK1/cyclin B1 pathway in nasopharyngeal carcinoma cells. Cell Cycle.

[20] Fisher D, Krasinska L, Coudreuse D, Novak B (2012). Phosphorylation network dynamics in the control of cell cycle transitions. J. Cell Sci..

[21] Forester CM, Maddox J, Louis JV, Goris J, Virshup DM (2007). Control of mitotic exit by PP2A regulation of Cdc25C and Cdk1. Proc. Natl Acad. Sci. USA.

[22] Lehmann BD (2016). Refinement of triple-negative breast cancer molecular subtypes: implications for neoadjuvant chemotherapy selection. PloS ONE.

[23] Song M (2016). Bystander autophagy mediated by radiation-induced exosomal miR-7-5p in non-targeted human bronchial epithelial cells. Sci. Rep..

[24] Cai S (2017). Exosomal miR-7 mediates bystander autophagy in lung after focal brain irradiation in mice. Int J. Biol. Sci..

[25] Karantza-Wadsworth V (2007). Autophagy mitigates metabolic stress and genome damage in mammary tumorigenesis. Genes Dev..

[26] Shang ZF (2010). Inactivation of DNA-dependent protein kinase leads to spindle disruption and mitotic catastrophe with attenuated Chk2 phosphorylation in response to DNA damage. Cancer Res..

[27] Maejima Y, Isobe M, Sadoshima J (2016). Regulation of autophagy by Beclin 1 in the heart. J. Mol. Cell. Cardiol..

[28] Xiao J (2015). FBXL20-mediated Vps34 ubiquitination as a p53 controlled checkpoint in regulating autophagy and receptor degradation. Genes Dev..

[29] Eskelinen EL (2002). Inhibition of autophagy in mitotic animal cells. Traffic.

[30] Pimkina J, Humbey O, Zilfou JT, Jarnik M, Murphy ME (2009). ARF induces autophagy by virtue of interaction with Bcl-xl. J. Biol. Chem..

[31] Liu WT (2014). MJ-66 induces malignant glioma cells G2/M phase arrest and mitotic catastrophe through regulation of cyclin B1/Cdk1 complex. Neuropharmacology.

[32] Huang R, Zhou P (2019). Double-edged effects of noncoding RNAs in responses to environmental genotoxic insults: Perspectives with regards to molecule-ecology network. Environ. Pollut..

[33] Zhou PK, Huang RX (2018). Targeting of the respiratory chain by toxicants: beyond the toxicities to mitochondrial morphology. Toxicol. Res. (Camb.).

[34] Mo LJ (2018). Exosome-packaged miR-1246 contributes to bystander DNA damage by targeting LIG4. Br. J. Cancer.

[35] Li H (2016). Atg5-mediated autophagy deficiency in proximal tubules promotes cell cycle G2/M arrest and renal fibrosis. Autophagy.

[36] Jia W (2015). Autophagy regulates T lymphocyte proliferation through selective degradation of the cell-cycle inhibitor CDKN1B/p27Kip1. Autophagy.

[37] Xu P (2017). Parvovirus B19 NS1 protein induces cell cycle arrest at G2-phase by activating the ATR-CDC25C-CDK1 pathway. PLoS Pathog..

[38] Lee JG (2013). Jaceosidin, isolated from dietary mugwort (Artemisia princeps), induces G2/M cell cycle arrest by inactivating cdc25C-cdc2 via ATM-Chk1/2 activation. Food Chem. Toxicol..

[39] Vera J (2015). Chk1 and Wee1 control genotoxic-stress induced G2-M arrest in melanoma cells. Cell. Signal..

[40] Qin J (2018). LncRNA Uc.173 is a key molecule for the regulation of lead-induced renal tubular epithelial cell apoptosis. Biomed. Pharmacother..

[41] Qin J (2018). LncRNA MIR31HG overexpression serves as poor prognostic biomarker and promotes cells proliferation in lung adenocarcinoma. Biomed. Pharmacother..

[42] Shen L (2018). LncRNA lnc-RI regulates homologous recombination repair of DNA double-strand breaks by stabilizing RAD51 mRNA as a competitive endogenous RNA. Nucleic acids Res..

[43] Jin F (2017). Inhibitory effect of uranyl nitrate on DNA double-strand break repair by depression of a set of proteins in the homologous recombination pathway. Toxicol. Res. (Camb.).

[44] Li M (2017). PIG3 promotes NSCLC cell mitotic progression and is associated with poor prognosis of NSCLC patients. J. Exp. Clin. Cancer Res..

