# Supplementary figures and images for: BECN1 promotes radiation-induced G2/M arrest through regulation CDK1 activity: a potential role for autophagy in G2/M checkpoint
Source: Cell Death Discov. 2020 Aug 5;6:70. doi: 10.1038/s41420-020-00301-2 (PMC7406511; doi:10.1038/s41420-020-00301-2)

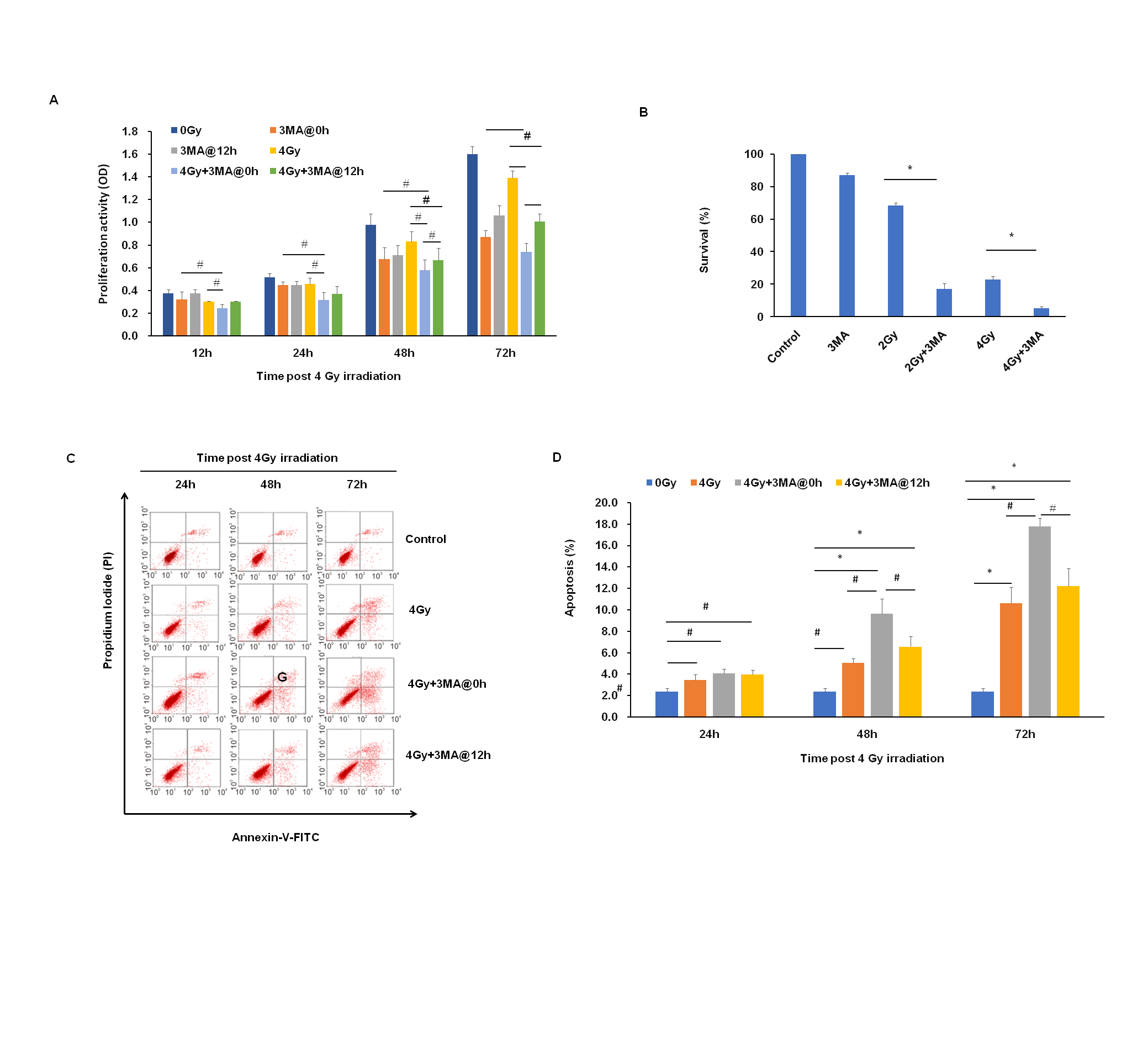

Supplement: Supplementary file 1 — Supplementary figure1 [file 41420_2020_301_MOESM1_ESM.tif]

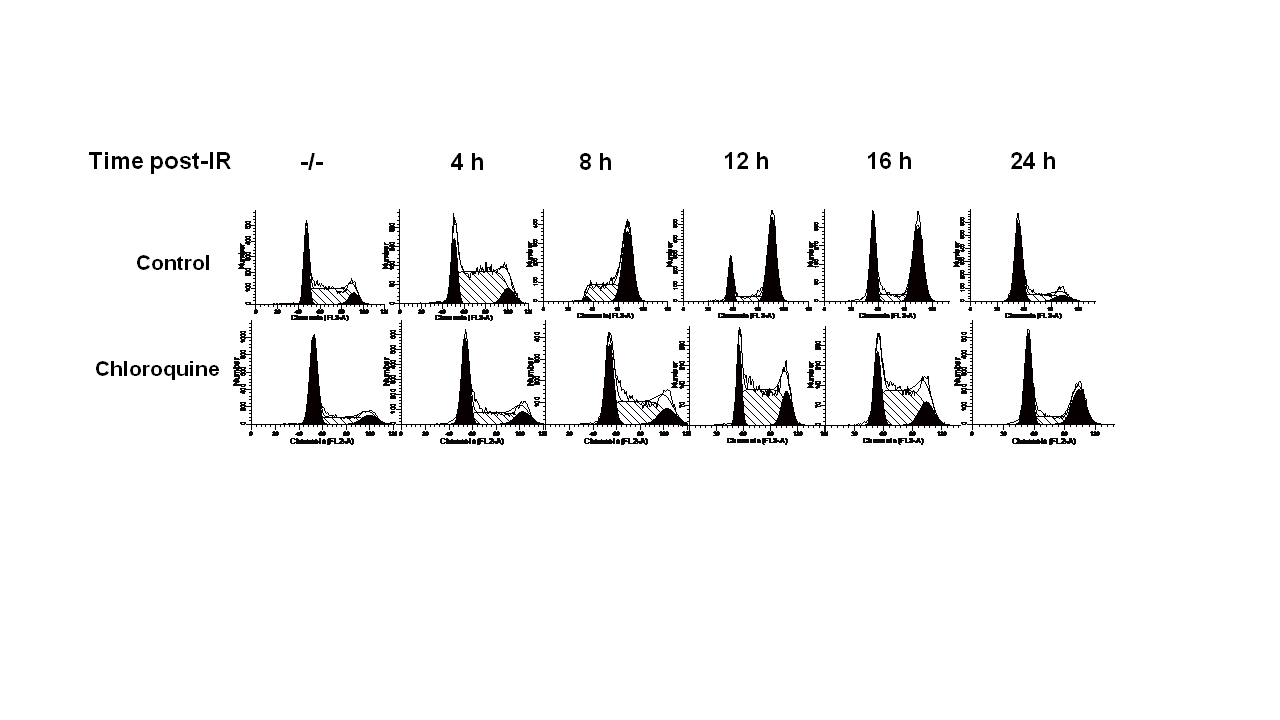

Supplement: Supplementary file 2 — Supplementary figure2 [file 41420_2020_301_MOESM2_ESM.tif]

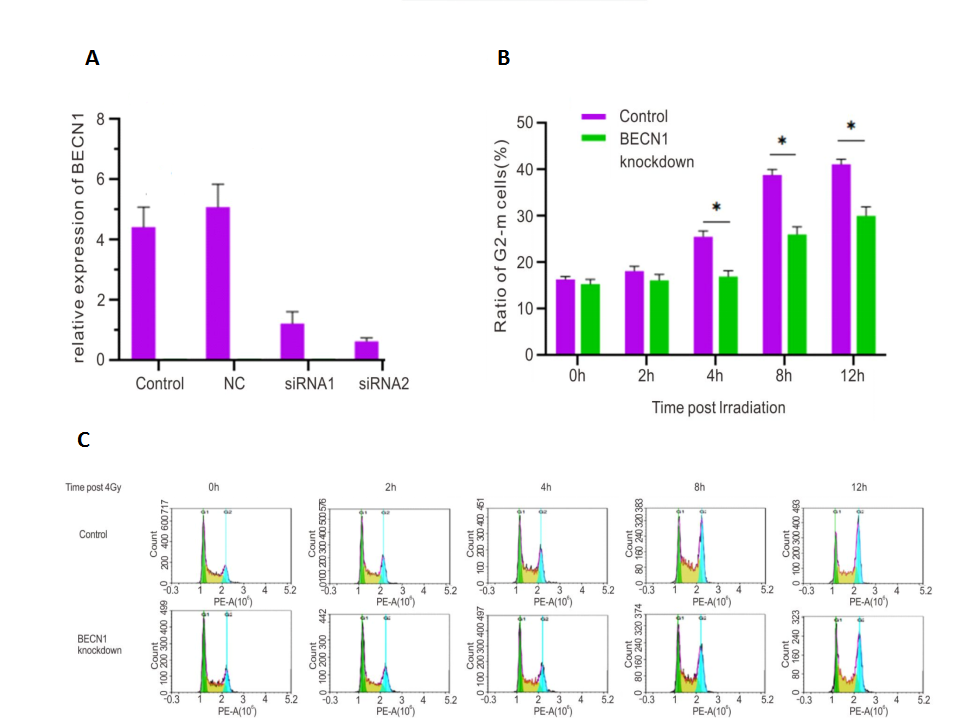

Supplement: Supplementary file 3 — Supplementary figure3 [file 41420_2020_301_MOESM3_ESM.tif]

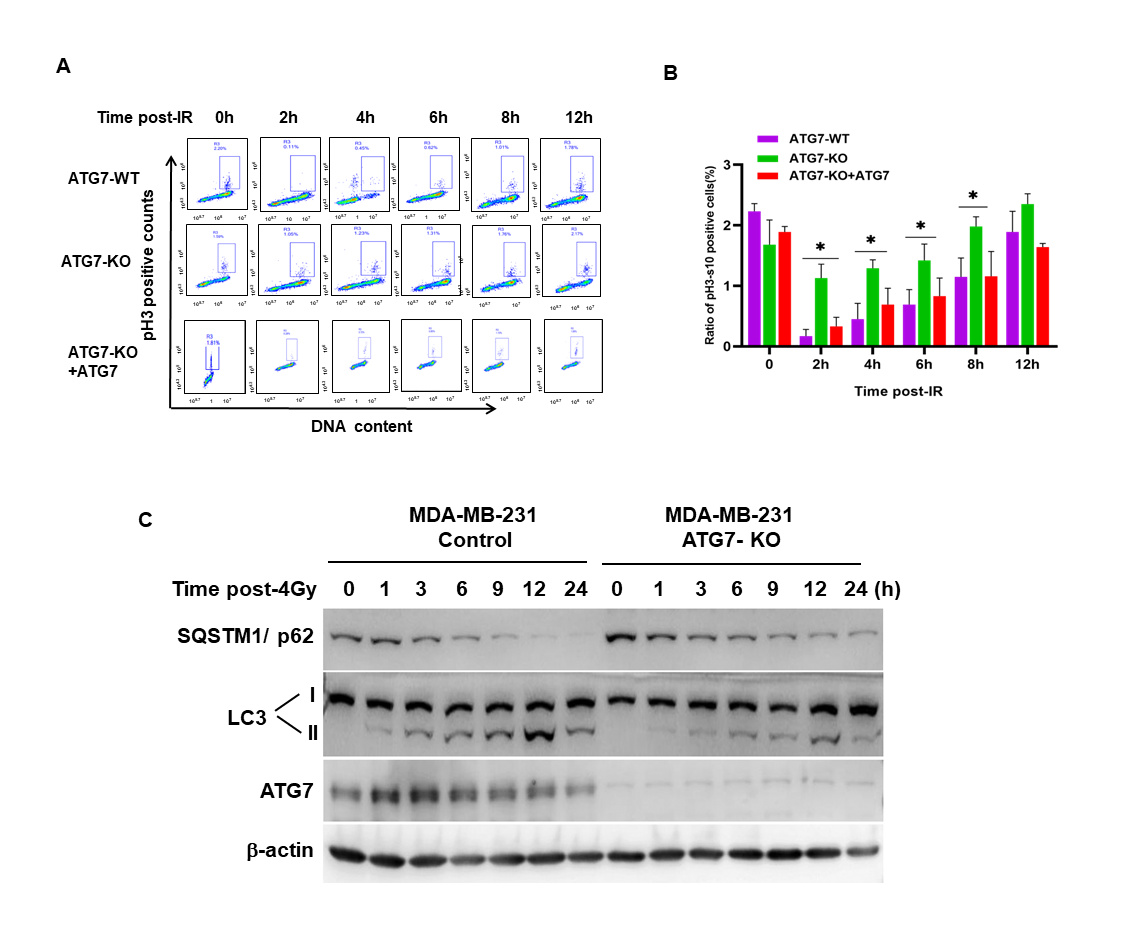

Supplement: Supplementary file 4 — Supplementary figure4 [file 41420_2020_301_MOESM4_ESM.tif]

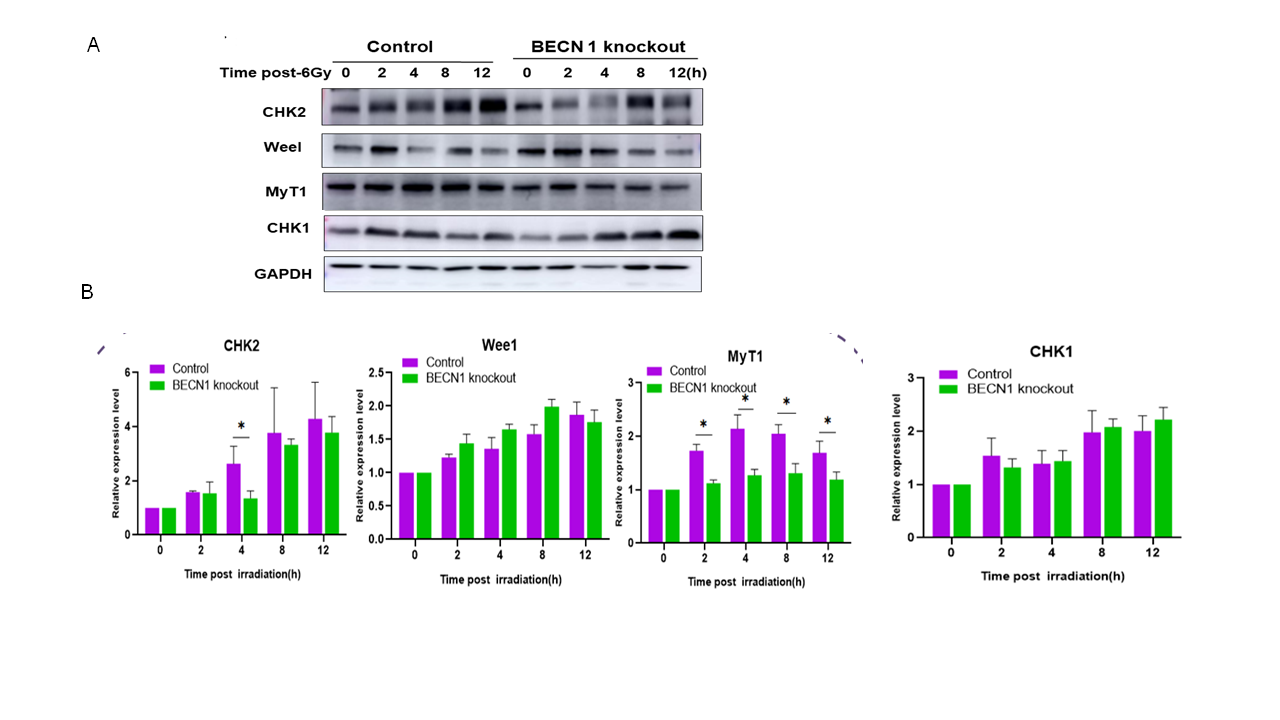

Supplement: Supplementary file 5 — Supplementary figure5 [file 41420_2020_301_MOESM5_ESM.tif]

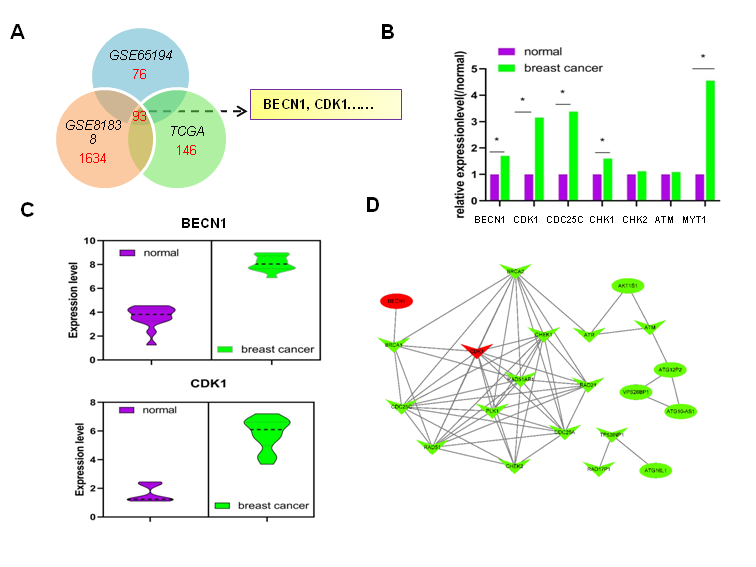

Supplement: Supplementary file 6 — Supplementary figure6 [file 41420_2020_301_MOESM6_ESM.tif]

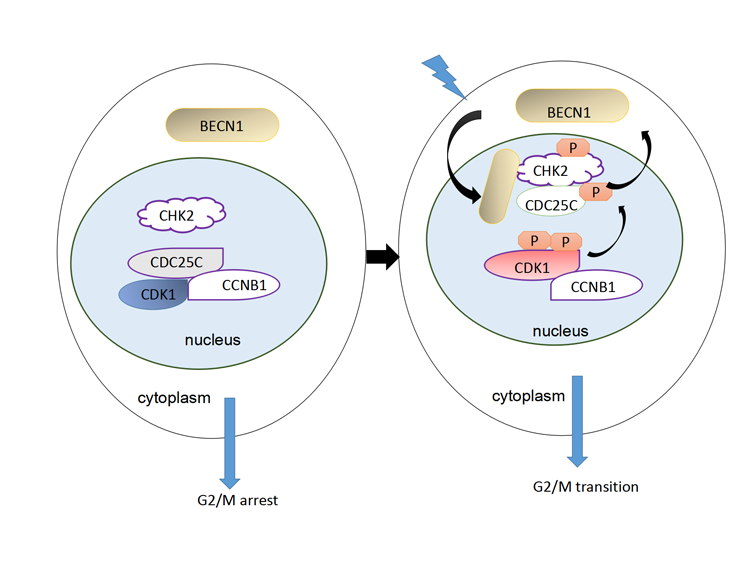

Supplement: Supplementary file 7 — Supplementary figure7 [file 41420_2020_301_MOESM7_ESM.tif]
